# Communication, Psychosocial, and Educational Outcomes of Children with Cochlear Implants and Challenges Remaining for Professionals and Parents

**DOI:** 10.1155/2011/573280

**Published:** 2011-09-05

**Authors:** Renée Punch, Merv B. Hyde

**Affiliations:** ^1^School of Education and Professional Studies, Griffith University, Gold Coast Campus, Gold Coast, QLD 4222, Australia; ^2^Faculty of Science, Education and Health, University of the Sunshine Coast, Maroochydore, Sunshine Coast, QLD 4556, Australia

## Abstract

This paper provides an overview and a synthesis of the findings of a large, multifaceted study investigating outcomes from paediatric cochlear implantation. The study included children implanted at several Australian implant clinics and attending a variety of early intervention and educational settings across a range of locations in eastern Australia. It investigated three major aspects of childhood cochlear implantation: (1) parental expectations of their children's implantation, (2) families' decision-making processes, and (3) the communication, social, and educational outcomes of cochlear implantation for deaf children. It employed a mixed-methods approach in which quantitative survey data were gathered from 247 parents and 151 teachers, and qualitative data from semistructured interviews with 27 parents, 15 teachers, and 11 children and adolescents with cochlear implants. The summarised findings highlight several areas where challenges remain for implant clinics, parents, and educators if children with cochlear implants are to reach their full potential personally, educationally, and socially.

## 1. Introduction

In most developed nations, the rate of paediatric cochlear implantation (CI) has increased to the point where it is now the predominant response to profound and, increasingly, severe childhood deafness. The majority of published studies investigating CI in children have focused on children's audition, speech production and perception, and spoken language development. As time goes on, it is becoming more possible to report on longer-term outcomes of children's functioning in their everyday lives at home, at school, and in the community [1, 2]. 

It is important to assess how children are functioning, communicating, and achieving in their everyday lives from parent and teacher reports, which may more closely reflect the functional outcomes of children in everyday life situations than assessments made in clinical settings [3–5]. The reported variability in outcomes among children with cochlear implants [6–9] adds to the importance of examining how implanted children fare over time, not only in their spoken language communication, but in their personal, social, and educational lives. 

The current study explored these broader areas of functional communication, educational, and psychosocial outcomes, as well as parents' preimplant expectations and postimplant experiences and the degree of concordance between these anticipated and experienced outcomes. In addition, it investigated parents' experiences of making the decision to choose CI for their children, and their experiences of the rehabilitation demands involved after their children's implantation. It utilised a mixed-methods approach that allowed quantitative survey findings to be extended and elaborated by qualitative findings from in-depth interviews. It included children with varying lengths of time since implantation, enabling insights to be gained into the lived experience and functional outcomes for children over time. 

The various aspects of the study and its findings have been reported in detail in papers covering the following topics: parents' experiences of making the decision for CI for their children [10]; parental expectations and experiences of their children's outcomes with CI [11]; educational supports and settings and teachers' perspectives of children's outcomes [12]; children's social participation from the perspectives of parents, teachers, and children with CI [13]; modes of communication and the role of sign language in the lives of children with CI [14]; factors predicting children's functional outcomes [15]; parents' experiences of efforts and stress involved in their children's CI and ongoing rehabilitation [16]. This paper provides an overview of the study and a synthesis of its main findings, points out challenges that remain for implant clinics, parents, and educators, and offers recommendations for professionals working with children receiving CI and their families.

## 2. Materials and Methods

A combination of quantitative and qualitative approaches was adopted in a sequential approach in which one method is used to further explore and expand the findings of another [17]. Quantitative data were derived from parents' and teachers' responses to a survey instrument, and qualitative data were derived from semistructured interviews with a subsample of the parent and teacher survey respondents, and from a small number of child and adolescent CI users. 

### 2.1. Participants

Survey participants were from two groups in three states of eastern Australia: (a) parents of children who had received cochlear implants before the age of 18, and (b) teachers working with deaf children in early intervention programs, primary schools, and secondary schools. Each teacher was asked to complete a survey based on one randomly selected child whom they supported.

Completed surveys used in the analysis numbered 247 for parents and 151 for teachers. Copies of the paper surveys and invitations to participate in the study were disseminated by a large number of schools, cochlear implant clinics, early intervention centres, and parent organizations for deaf children, and we cannot know exactly how many parents or teachers received a copy of the survey or an invitation to complete the survey. Figures provided by the cochlear implant clinics indicated that 1260 children under the age of 18 had been implanted up to the data collection period in March 2008. However, there is no way of knowing what percentage of that number of families received the survey or invitation to participate.

Of the parent survey respondents, almost 90% were mothers. Almost 97% were hearing, with 7 parents stating that they were hard of hearing and one parent identifying as Deaf (capital D used to denote identity with a Deaf community). Most of the teachers worked as itinerant teachers of the deaf who visit students and collaborate with class teachers in regular school settings (40%), or as teachers of the deaf based on a support class or deaf facility in a regular school (40%); the remainder were largely early intervention specialists or general class teachers.

Approximately 25% of the children in both the parent and teacher surveys had additional disabilities or difficulties. Respondents specified a wide range of conditions including learning difficulties, cerebral palsy, intellectual impairment, asthma, and needing to wear glasses. Tables 1 and 2 show further details about the children, including their age at data collection, age at implantation, and educational setting.

Although parents and teachers were reporting on children drawn from the same population (i.e., all children who had been implanted in New South Wales, Victoria, and Queensland), these two groups of respondents were not necessarily reporting on the same children in each case.

Approximately 10% of the survey respondents in these two groups were interviewed: 27 parents and 15 teachers. In addition, 11 CI users, aged between 10 and 17 years, were interviewed.

### 2.2. Measures

The parent survey contained three sections. The first collected background information, including family demographics and child-related information such as age at hearing loss occurrence and identification, age at cochlear implantation, occurrence of bilateral implantation, use of hearing aids before the implant (and since, if used in the nonimplanted ear), educational setting, and communication approach in the educational setting. The second section asked about the process of making the decision about CI, and included questions about the sources of information parents used while making the decision to have their child implanted, the length of time parents considered CI before making their decision, and their satisfaction with the information, help, and support received from a range of professionals.

The third section asked about parents' expectations and experiences of their children's CI and was adapted from a survey developed by Zaidman-Zait and Most [18] for use with mothers of children with CI. For the current study, modification was made to some of the items to more closely reflect the Australian context, and a small number of items was added. This section of the survey contained seven subscales. Five of these asked about the outcomes that parents had expected and experienced in their children with CI. They were (1) communication abilities, which reflected abilities in spoken language in functional, everyday situations; (2) social skills and participation, which was concerned with children's acceptance by hearing peers, social participation with children in general, and social skills; (3) well-being and happiness, with items about children's happiness, frustration, and safety with the implant; (4) academic achievements, including children's ability to participate in regular classes and items concerning levels of achievement in numeracy and literacy; (5) future life, with items about children's general functioning and independence as well as their identity as deaf or hearing persons. 

Two subscales (rehabilitation efforts and rehabilitation stress) asked parents about the demands on themselves and their families of the ongoing rehabilitation process and their perceptions of stress around making the decision to implant, the rehabilitation process, and generally encountered with their deaf children.

For each item on all subscales, parents were asked about their expectations before their child received the CI and about their experiences currently, since their child had the CI. In addition, six items rated parents' overall satisfaction with their children's social, academic, and communication abilities; for example, “overall the expectations I had before my child was implanted are now being met” and “currently, I am satisfied with my child's communication abilities.” Respondents were asked to rate their level of agreement with each item on a 5-point scale with responses *strongly disagree, disagree, neither agree nor disagree, agree* and *strongly agree*. At the end of the survey, parents were invited to write an open-ended response to the question “if there is one central message that you would like to convey to us about the experiences you have had with your deaf child and his or her cochlear implantation, what would that be?” 

The teacher survey also contained 3 sections. The first section collected data on the role of the teacher (e.g., itinerant support teacher, early intervention specialist, support class teacher, and general class teacher), and details about the child, including gender, current age, age at implantation, occurrence of bilateral implantation, hearing aid use, type of educational setting, and the communication approach or program used with the child in that setting.

Section 2 asked teachers to report on outcomes in the same domains as on the parent survey, with the exclusion of the wellbeing and happiness subscale, which contained items specific to parents' knowledge of their children.  Section 3 assessed the level of participation of children in their school and classroom activities according to a framework devised by Mirenda [19]. The framework covers four aspects of participation in regular classrooms: integration, described as physical presence in the regular classroom; academic participation; independence, which is concerned with the level of supports the child needs in order to participate in the classroom; social participation. This framework has been used effectively in another Australian study with deaf and hard of hearing students [20]. At the end of the survey, teachers were invited to write any additional comments they wished to make.

### 2.3. Procedure

Approval for the project was gained from the relevant university, state government education department, early intervention centre, and hospital Human Research Ethics Committees. Cochlear implant clinics, early intervention centres, and the education departments facilitated the distribution of copies of the survey to all families and teachers of implanted children on their data bases. Parents and teachers were informed of the option of completing and submitting the questionnaire online. All survey and interview data were collected in 2008.

### 2.4. Interviews

We aimed to conduct follow-up interviews with approximately 10% of parents and teachers who completed surveys. Because almost 80% of both the parent and teacher survey respondents agreed to be contacted for an interview, we needed to make a selection of participants to invite to be interviewed. In keeping with the aims and qualitative approach of this phase of the study, sampling was purposeful, that is, designed to select information-rich cases likely to best illuminate the questions being investigated and yield insights and in-depth understanding, rather than empirical generalizations [21]. We selected a range of parents and teachers in terms of location (metropolitan, regional, rural), current age of child, age of child at implantation, and the type of educational setting the child attended, so that there would be structured representation across the range of situations of children with CI. 

The semistructured interviews used an initial list of questions serving as a guideline only, allowing unanticipated information to emerge. The questions included in the interview guide covered the parents' decision making about CI for their children, their expectations before their children's implantation, and their experiences and perceptions of their children's communication, personal, social, and educational development since implantation. In the teacher interviews, the questions fell into two categories: first, questions that related to the specific child about whom the teacher had completed the survey and, second, questions relating more generally to the teachers' experiences of working with children with CI. Teachers were asked about their experiences of the outcomes of CI in terms of the child's development in language and communication, educational achievement, social participation, and identity. Children and adolescents were asked open-ended questions about their feelings about their cochlear implants, their communication modes, their use of telecommunications technology, and their friendships. Further details about the parent, teacher, and child interviews are presented in the individual reports emanating from the larger study [10–14, 16].

## 3. Results and Discussion

### 3.1. Making the Decision for Cochlear Implantation

A full account of the quantitative and qualitative findings in regard to parents' decision making is presented in Hyde et al. [10]. The quantitative findings indicated that, in making the decision for their children to have CI, parents used a range of sources of information. Cochlear implant programs and audiologists were the most frequently used sources. Families of children with CI were also a major source of information for parents. Relatively few parents used deaf organisations or deaf adults, with or without CI, as information sources. In the interviews, many parents said that they would have liked to have received information on a broader range of subjects, including social and educational aspects and communication methods, than was made available to them at the time of making the decision.

Overall, the decision-making period was quite short, with 60% of parents taking less than three months to make the decision. Almost half (48%) of the parents reported that making the decision to have their child implanted was extremely stressful. However, a sizeable group of parents (39%) reported otherwise. The qualitative findings showed that some parents decided quickly, usually because they felt that an implant was the only way their child would gain communication through speech and hearing, and so was the only option for their child. Other studies have reported that deciding about CI was easier for parents who believed their children would not develop speech without an implant [22] and who placed the most importance on oral communication rather than signing [23, 24].

### 3.2. Parents' Expectations and Experiences of Children's Outcomes

The study compared parents' reports of their preimplant expectations with their experiences of postimplant outcomes on a large number of items related to five outcome domains [11]. The quantitative findings indicated that this group of parents had held relatively high expectations of their children's communication, social, academic, wellbeing, and future life outcomes from CI. These findings are consistent with reports in the literature of high parental expectations [18, 25, 26]. However, our findings did not reflect the almost uniformly high expectations of the mothers in Zaidman-Zait and Most's study [18]; rather, more variability in expectations was found among our larger sample. 

In addition, the findings indicated that parents' experiences of their children's outcomes with CI were at relatively high levels. In most outcome domains, there was no statistically significant difference between parents' expectations and subsequent experiences of their children's outcomes with the CI. On some items in the subscales, there were high levels of uncertainty in expectations; for example, 35% were uncertain whether their child would easily make friends with hearing children, and 23% were unsure if their child would be able to use the telephone. Uncertainties were informed by subsequent experiences, usually in a positive direction. 

In response to questions about overall satisfaction with the CI, approximately four fifths of the parents indicated that their expectations had been met. These findings suggest that the majority of parents had high levels of satisfaction with their children's outcomes with their CI. However, a tenth of parents reported that their overall expectations had not been met. Further, a tenth of parents were not currently satisfied with their children's communication abilities, social skills, and academic abilities. These findings show consistency with those of other studies in which between 5% and 20% of parents have reported unmet expectations [27, 28]. Our study's qualitative findings, through both the interview data and survey open-ended responses, indicated that the parents who were disappointed tended to be those whose children had additional disabilities or conditions that precluded them from gaining a great deal of benefit from their implants. This was particularly the case when the child was implanted at a young age and the additional conditions were unknown until some time after implantation, and parents' expectations of good spoken language development were not realised.

From the qualitative findings, it is clear that parental expectations were closely intertwined with parental hopes. The findings suggest that parents' levels of expectations, beliefs, and hopes about CI may reflect their determination to do the best for their child and their conviction that a CI is the only way for their child to most fully participate in a hearing world, rather than the explanations and cautions about potential outcomes that may be presented to them by professionals. The parents tended to interpret such explanations in the most positive context.

The retrospective nature of parents' responses about their expectations prior to their children's implantation may have the potential to be affected by recall bias. However, in other studies, researchers have found that most parents had detailed and accurate recall of significant events such as the diagnosis of their children's deafness even many years later [29, 30]. We found in the interviews that the stories of discovering their children's deafness and their subsequent thoughts, feelings, and actions were vivid in the minds of the parents, who showed clear recall of the period leading to their children's implantation.

### 3.3. Children's Outcomes

This section provides a summary of the quantitative findings of children's outcomes from each of the communication, social, academic, wellbeing, and future life domains in the study, as well as the qualitative findings pertaining to these outcome areas. Full details are presented in the relevant individual papers [11–13].

#### 3.3.1. Communication Outcomes

Spoken language communication abilities were reported to be relatively high by parents and somewhat lower by teachers. Some children were developing, or had developed over a period of years, near normal speech and language outcomes, but these tended to be in specific situations involving familiar communication partners, such as family members, and in familiar contexts and optimal listening environments. In broader contexts, such as in regular classrooms and in social groups and gatherings, the picture was not as positive, with parents and teachers reporting difficulties for children in these environments. For instance, 20% of parents and 48% of teachers reported that the child could not follow a spoken conversation with a group of people. These findings were confirmed in the qualitative findings in which interviewees described difficulties in groups of hearing peers.

#### 3.3.2. Social Outcomes

In the survey subscale social skills and participation, parents indicated relatively positive outcomes for their children. However, the qualitative findings revealed that the area of their children's social skills and participation remained a concern for most parents, who were aware of their children's difficulties in groups and how those difficulties affected their social inclusion. In addition, findings from both the quantitative and qualitative teacher data indicate less than optimal social outcomes. For example, teachers reported that a third of the children did not easily make friends with hearing children and did not have age-appropriate social skills, and 10% were not accepted by their hearing peers. The findings from the interviews with parents, teachers, and adolescents indicated that situations involving hearing peers were often difficult, and the development of some social skills, such as understanding subtleties and nuances in social interactions, were delayed in these children with CI. Social participation and emotional wellbeing became more problematic for some children as they reached adolescence and appeared to struggle with issues around being deaf, feeling self-conscious about the CI external equipment they needed to wear, and fitting in with hearing peers. These findings are similar to others reported in studies on social participation and quality of life for adolescents who use hearing aids [31, 32] and cochlear implants [33] and are consistent with the findings of Martin et al.'s study [34] in which children with CI performed better interacting with hearing peers in one-on-one situations than in interactions including two hearing children. It appears that even children with excellent outcomes in spoken language development and communication experience the phenomenon of “social deafness” [35], a term used to describe the effects of hearing loss in social interactions involving groups of people or in noisy environments, in contrast to one-on-one interactions, which are generally easier for people who are deaf or hard of hearing to manage.

#### 3.3.3. Academic Outcomes

More than two thirds of parents reported that their children were able to participate easily in a regular class, and slightly more than one third of teachers reported this. Between 50% and 60% of teachers disagreed that children were achieving high standards in reading, writing, and maths or were achieving at the expected level for their age, whereas between 18% and 23% of parents indicated disagreement on these items. Almost 70% of children in the teacher reports fell below the class median in academic performance.

Although the teachers interviewed were generally positive about the outcomes and educational experiences of students with cochlear implants, they identified a number of problems and challenges. A major concern was that some students were at risk of missing out on learning in certain pedagogical environments such as group discussion activities, and that often students would not admit their difficulties and seek help in those situations. This is a challenge for educators, particularly in secondary schools, where children who have done well in primary school may need more specialist support to access a more challenging curriculum [36]. However, deaf adolescents, not wanting to be seen as different, can resist what they see as the stigma of being singled out for assistance by itinerant teachers or other support services. In our qualitative findings, teachers reported that some secondary school students with CI were reluctant to use FM systems and other supports in school.

Parents and teachers often used the term “still deaf” to describe their children and asserted that many people, including regular class teachers and school authorities, had misconceptions about the nature of CI. These parent and teacher interviewees believed that there was a lack of understanding that CI does not “fix” deafness and that children with CI still experience difficulties in many auditory environments and delays in aspects of their development that necessitate ongoing support and accommodations in the school setting. It appeared that this lack of understanding was particularly the case for those children whose spoken language was good. Other studies have reported a similar lack of understanding on the part of teachers (and other students) about students with hearing loss in regular classes [37, 38].

Some of the teachers interviewed reported a lack of liaison with CI clinics and of professional development about CI for themselves as well as for regular class teachers. Teachers pointed out the complexity of the technology involved, especially when cochlear implants are used with FM systems and hearing aids, and the challenges for teachers and parents attempting to identify and rectify problems with the equipment. Other researchers have emphasized the importance of close liaison between CI centres and local educational services in order to ensure the best management and continuing use of the CI technology [27, 39].

#### 3.3.4. Wellbeing and Future Life Outcomes

In areas of children's general functioning, independence and identity as deaf or hearing persons, the large majority of parents believed their children were happier, less frustrated, and safer than they would have been without the implant. Almost 60% of parents believed their child functioned like a child with normal hearing, whereas only 30% of teachers reported this. In terms of identity, close to 30% of parents and teachers believed the child had developed an identity as a deaf person, while two thirds of parents, and slightly more than one third of teachers, reported that the child comfortably shared both deaf and hearing identities. These findings are consistent with those of implanted adolescents' self-reported sense of identity in other studies. For example, a UK study found that young people displayed a flexible attitude towards their identity as deaf or hearing people [40], and a US study reported that, although a group of adolescents with CI were more acculturated to hearing society than a group of deaf adolescents without CI, who were more Deaf acculturated, 40% of both groups indicated a bicultural (deaf/hearing) identity [41]. The qualitative findings in the current study indicated a perception by teachers of a move towards more of a deaf or bicultural identity by some students during their adolescence. 

### 3.4. Impact on the Family, Rehabilitation Demands and Parental Stress

The findings related to rehabilitation demands and parental stress are reported in detail in Punch and Hyde [16]. A statistically significant difference was found between parents' expectations and experiences on the rehabilitation stress subscale of the survey (*t* = −2.41 (df = 246), *P* < 0.05), with parents experiencing more stress than they had expected before their children's CI. A major theme in the qualitative findings concerned the amount of work and time parents dedicated to their deaf children in the areas of early intervention and speech training as well as in terms of frequent appointments for mapping and other ongoing requirements related to the use of the CI. They reported feeling stressed by the time needed to travel to and attend appointments, and by the difficulties this imposed on their other children and on the family's financial situation. Parents described frequent problems with implant equipment breaking and parts needing replacing. When these could not be replaced quickly, it was worrying for parents and difficult and frustrating for the children to be unable to use their implant and thus be without their means of hearing until replacements arrived. Similar concerns have been reported in studies of parental experiences of their children's CI [27, 28, 42, 43].

A recurring theme throughout the qualitative data was of the difficulties and stress experienced by parents living some distance from implant, rehabilitation, and early intervention services. In Australia, CI is undertaken in hospitals in major cities only, and most of the providers of early intervention services for deaf children are based in the major cities although some have centres or therapists in regional areas and some provide an outreach service for remote families. However, some families live large distances from many of the services they need for their deaf children. Spencer [44] reported similar problems for some of the Australian families of children with CI in her study. 

Parents whose children did not do well with the implant and whose speech and language development was poor went through particularly stressful periods. In cases where auditory-oral approaches were not proving effective and parents moved towards using sign, they often lost a valuable support base if they were unable to continue attending their auditory-oral early intervention centre. 

### 3.5. Communication Modes and the Role of Signed Communication in the Children's Lives

One aim of the study was to ascertain the extent of use of various forms and modes of communication, including oral-aural, sign-supported spoken English, and Australian sign language (Auslan), by children with CI, both in their educational settings and with their families. The findings in regard to children's communication patterns and use of signed communication are reported in full in Hyde and Punch [14]. The quantitative findings indicated that parents overwhelmingly chose CI for their children as a mean to develop communication through hearing and speaking, and that parents' expectations in the main were that their children would not need to use a sign language or sign support. However, experience seemed to have tempered this perspective to some extent, and a substantial proportion (between 20% and 30%) of children were reported by both teachers and parents to be using some form of signed communication.

The qualitative findings elaborated on these findings, providing details of ways in which children were using signed communication. The interview findings showed that many parents had become convinced of the benefits of signing for their children. Of the 27 parents interviewed, 19 were using signed communication to some extent with their children. Some parents found that early use of signing aided their children's spoken language development after CI. In addition, parents saw the value of developing communication with their children through signing so that they could communicate whenever the children were not wearing their implants' external device, whether because of equipment breakdown or when the children were in situations where it could not be worn, such as swimming. Several of the parents also valued Auslan as a way for their children to establish a connection to other deaf people and a sense of Deaf identity. Overall, these findings show consistency with studies from several countries reporting that signed communication is used by many implanted children, usually as a support to their spoken language acquisition, and is not incompatible with the main aim of the development of oral communication [45–48].

### 3.6. Factors Predicting Outcomes

Associations among the four outcome variables communication abilities, social skills and participation, academic achievements, and future life (average subscale scores) and a select array of variables were examined via a series of step-wise regression analyses of the parent survey data. These findings are presented in full in Hyde et al. [15]. In addition to considering a number of important factors examined in other studies, the present study included a number of independent variables not normally included in regression analyses in paediatric CI studies, such as parental expectations, length of time making the decision to implant, and families' geographic location. Among the large number of variables related to family and child characteristics, educational and communication factors, and the parents' implant decision-making process entered into the regression analyses, a number of key predictors of the outcome domains were identified.

Several variables related to oral communication and mainstream placement were shown to predict positive outcomes in many of the outcome domains. The outcomes of children who were more “oral” were rated more highly by their parents on the everyday functioning areas of hearing and spoken language communication, social skills and participation, and general functioning and independence, but not in the area of academic achievements. The child being in regular, mainstream educational settings was predictive of positive communication and academic outcomes. In the area of communication abilities, these findings are consistent with other reports [49–53]. However, it is not possible to know whether being in a mainstream setting and following a spoken language communication approach was a cause or a consequence of good spoken language or academic abilities. In Australia, sign language programs are largely associated with the presence of additional disabilities, and there is a scarcity of comprehensive bilingual programs outside capital cities. This combination of factors means that many of the students deemed to require access to sign language or signed English were placed in special education rather than regular class programs; in these contexts, a high standard of oral communication outcomes is not the norm. The study found no negative predictor values for parents' use of signed communication or the use of signed communication in the educational setting. Further research examining the impact of bimodal and bilingual communication with deaf students with CI is necessary.

Consistent with other studies [54–56], a younger age at implantation was found to be a predictor of positive communication and social outcomes. The child having a bilateral implant was predictive of positive communication, social, and academic outcomes, noteworthy findings adding to the still relatively limited knowledge of broader outcomes of bilateral paediatric implantation. All bilaterally implanted children in the current study had received their bilateral implant sequentially, rather than simultaneously.

The child having additional disabilities was strongly predictive of less positive outcomes in all domains. We cannot know from the data whether additional disabilities limited the benefits of the children's CI or whether they affected the children's ability to achieve positive outcomes, regardless of CI.

The regression findings indicated that families' location in major city areas was associated with positive communication outcomes, and that living in regional or remote areas predicted greater rehabilitation stress for parents. 

## 4. Challenges and Implications for Professionals

This large, multifaceted mixed-method study's findings have highlighted challenges that remain for cochlear implant programs, support services, early intervention providers, education authorities, teachers, and families and have implications for professionals working with families and children who have CI. The major challenges, implications, and recommendations that the study's findings have suggested are as follows.

Implant programs should continue to advise with caution about the range of likely outcomes but also be aware that families are likely to be influenced by their hopes and aspirations for their children as much, if not more, than by the information they have received. Consequently, information-giving processes should be regularly repeated, extended, and evaluated through ongoing discussion and counselling. 

There should be greater use of the experiences of deaf and hard of hearing people in support of the decision-making process, and of associated media materials available for those parents not able to attend day or evening sessions. Increased opportunity should be provided for parents to consider situations where their child may use signed communication or develop more than the identity of a “hearing” child. It is important for information about sign language and the Deaf community to be more accessible to families both before and after their children's implantation. Parents should be made aware that choice of a communication mode need not be an either/or option, and that sign language exposure or bilingualism is not solely something to be resorted to if children fail to develop oral-aural communication proficiency but can be used to provide fuller access to cognitive development and communication competence across a range of situations and social settings, thus maximising the child's life experience and potential. 

It is important for service providers to respond with flexibility when children's and parents' needs change over time, particularly when expected outcomes are not achieved, and it becomes apparent that alternative strategies and approaches are necessary.

Perhaps one of the most concerning aspects of the study's findings is that 70% of the children were judged by their teachers to be below the median level of their class in academic achievement, particularly in literacy and numeracy. While the parent data suggest that they were not as aware of this outcome as the teachers, there would seem to be an urgent need to address this situation if these children are to have school achievements commensurate with their communication potential and language acquisition. This remains a significant challenge, as Marschark et al. [57] indicated several years ago, and studies of the achievements of deaf and hard of hearing children in various curriculum areas remain very limited in number.

From the perspective of their functional communication, children with cochlear implants should be supported by teachers and school authorities essentially as if they were hard of hearing; that is, the children are likely to need supports similar to those provided to children without CI with moderate or severe levels of hearing loss. Even those children whose spoken language capacity and proficiency is high are likely to have listening difficulties in particular social and educational contexts and will not have full access to school curricula or to many activities promoting social inclusion. 

It is necessary for educators involved with children with CI, both teachers of the deaf and general classroom teachers, to have access to professional development and training about CI device equipment and the needs of children with CI. In addition, the maintenance of the CI device equipment necessitates strong communication links among teachers, parents, and implant professionals. 

The clear links found between families' localities and parental stress and children's communication outcomes suggest that, for children to receive optimal benefits from their implants and for the demands and stress on parents to be reduced, it is necessary that continuing efforts are made to improve access to audiological, rehabilitation, and ongoing device maintenance services for families who live away from major urban centres. 

In conclusion, the results of the study are largely positive and essentially reflective of parents' high hopes and expectations of CI for their children. Proponents of implantation, both professional and nonprofessional, are generally quick to accept and project these largely positive findings. However, they are often more reticent about supporting equally consistent findings, such as are reported in this and other studies, indicating that signed communication and sign languages will also be part of the lives of many of these children, with the potential to benefit their spoken language development as well as their social development and participation within their various communities. In some ways, the very success that these children seem to be demonstrating in early spoken language acquisition and use within their families has the potential to mask some of the difficulties that were found in this study within schools, the curriculum, and in some social settings. 

While making clear the benefits of CI and its important role in extending opportunities for profoundly deaf children, this mixed-method study's findings indicate that challenges remain for children with CI and families, implant clinics, early intervention centres, and educators. Systems that support children with CI and their parents face challenges most notably in the areas of children's academic achievement and social development and participation with hearing peers. Ongoing attempts to address these challenges are essential if children with CI are to be fully supported to reach their potential personally, educationally, and socially. 

## Figures and Tables

**Table 1:** Characteristics of children in the parent surveys (*N* = 247).

		*M*	Range	SD
Age at data collection (yrs)		9.42	.67–25.0	4.63
Age at implantation (yrs)		3.27	.38–16.42	3.16
Age at bilateral implantation (yrs; *n* = 65)		5.16	.63–18.42	4.09

Gender				
Male	49.8%			
Female	50.2%			

Educational setting				
Main-stream	21.8%			
Special education	47.3%			
Other	30.7%			

Locality				
MC	61.0%			
IR	29.3%			
OR/R	9.8%			

*Note*. MC: major city; IR: inner regional area; OR/R: outer regional and remote area.

**Table 2:** Characteristics of children in the teacher surveys (*N* = 151).

		*M*	Range	SD
Age at data collection (yrs)		10.37	1.33–18.67	4.56
Age at implantation (yrs)		4.11	.67–16.58	3.76
Age at bilateral implantation (yrs; *n* = 23)		5.92	1.08–15.83	3.91

Gender				
Male	44.4%			
Female	55.6%			

Educational level				
EI	21.9%			
1–6	40.3%			
7–12	37.1%			

Educational setting				
Main-stream	51.7%			
Special education	42.3%			
Other	5.3%			

*Note*. EI: early intervention centre; 1–6: school grades 1–6; 7–12: school grades 7–12.
